# Robust Output Feedback Stabilization and Tracking for an Uncertain Nonholonomic Systems with Application to a Mobile Robot

**DOI:** 10.3390/s24113616

**Published:** 2024-06-03

**Authors:** Muhammad Junaid Rabbani, Attaullah Y. Memon, Muhammad Farhan, Raja Masood Larik, Shahzad Ashraf, Muhammad Burhan Khan, Zeeshan Ahmad Arfeen

**Affiliations:** 1 Department of Electrical Engineering, National University of Computer and Emerging Sciences, Karachi 75030, Pakistan; burhan.khan@nu.edu.pk; 2 Department of Electronics and Power Engineering, PN Engineering College, National University of Sciences and Technology, Karachi 75500, Pakistan; attaullah@pnec.nust.edu.pk; 3 Department of Electrical Engineering and Technology, Government College University Faisalabad, Faisalabad 38000, Pakistan; mfarhan@gcuf.edu.pk; 4 Department of Electrical Engineering, N.E.D University of Engineering and Technology, Karachi 75270, Pakistan; rmlarik@neduet.edu.pk; 5 Department of Electrical Engineering, NFC Institute of Engineering and Technology, Multan 60000, Pakistan; 6 Department of Electrical Engineering, DHA Suffa University, Karachi 75500, Pakistan; 7 Department of Electrical Engineering, The Islamia University of Bahawalpur, Bahawalpur 63100, Pakistan

**Keywords:** backstepping control, high gain observer, nonholonomic wheeled mobile robot, sliding mode control, stabilization, trajectory tracking

## Abstract

This paper presents a novel robust output feedback control that simultaneously performs both stabilization and trajectory tracking for a class of underactuated nonholonomic systems despite model uncertainties, external disturbance, and the absence of velocity measurement. To solve this challenging problem, a generalized normal form has been successfully created by employing an input–output feedback linearization approach and a change in coordinates (diffeomorphism). This research mainly focuses on the stabilization problem of nonholonomic systems that can be transformed to a normal form and pose several challenges, including (i) a nontriangular normal form, (ii) the internal dynamics of the system are non-affine in control, and (iii) the zero dynamics of the system are not in minimum phase. The proposed scheme utilizes combined backstepping and sliding mode control (SMC) techniques. Furthermore, the full-order high gain observer (HGO) has been developed to estimate the derivative of output functions and internal dynamics. Then, full-order HGO and the backstepping SMC have been integrated to synthesize a robust output feedback controller. A differential-drive type (2,0) the wheeled mobile robot has been considered as an example to support the theoretical results. The simulation results demonstrate that the backstepping SMC exhibits robustness against bounded uncertainties.

## 1. Introduction

In recent years, nonholonomic systems have been considered a challenging benchmark problem due to their restricted mobility and underactuated behavior. These systems satisfy non-integrable velocity constraint, which originates as a result of physical kinematic constraints (such as pure rolling conditions) imposed on the system. This classical subject has gained significant attention due to the prevalence of nonholonomic constraints in numerous advanced robotic structures, including space manipulators, legged robots, wheeled mobile robots (WMRs), and multi-fingered robot hands.

WMRs are a prime example of nonholonomic dynamic systems and are commonly used as a benchmark problem for this class of systems due to their limited mobility and underactuated behavior. The two main control tasks of WMRs are tracking to a reference trajectory and posture stabilization at the desired pose [1]. From a control perspective, posture stabilization of a mobile robot is a more challenging task than trajectory tracking. This is because posture stabilization is considered an input-state problem, with two control inputs responsible for zeroing three independent configuration errors ($e_{x},e_{y},e_{\theta }$). On the other hand, trajectory tracking can be recognized as an input–output problem, with two control inputs available to zero two-dimension errors ($e_{x},e_{y}$). In fact, it is understood that these systems cannot achieve asymptotic stabilization via smooth time-invariant feedback control [2].

In the literature, many researchers have proposed solutions for trajectory tracking and posture stabilization using kinematic models [3,4,5,6,7]. However, few research papers have focused on integrating mobile robot kinematic and dynamic models without considering the parameter uncertainties and external disturbances [8,9,10]. Input–output feedback linearization has been widely utilized to develop feedback controllers for WMRs due to its challenging nonlinear model [1,5,7,11,12,13]. These papers use the exact model of WMR for the cancellation of nonlinear terms that may cause inaccuracy during trajectory tracking and posture stabilization. However, in practical scenarios, the exact knowledge of kinematic and dynamic model parameters is not known due to uncertainties. Therefore, robust/adaptive-based tracking and stabilization controllers have been proposed to mitigate the effect of external disturbances and model uncertainties [14,15,16,17,18]. In particular, SMC is preferred because of its robustness against parameter uncertainties and external disturbance, satisfactory transient behavior, and fast response [19,20,21,22,23,24]. It is also notable that most of these papers have studied stabilization and tracking problems separately.

Another major issue found in a mobile robot is the measurement of velocity components and other states through sensors. However, some states are difficult to measure due to the cost, weight limitation, and sensor unavailability. Furthermore, in practical situations, velocity measurement using a sensor may degrade the desired performance due to the measurement noise. For that reason, output feedback control is the most effective solution to avoid sensor-based measurement of velocity components. In [3,15,25,26,27,28,29,30], trajectory tracking and stabilization [31,32,33] based on output feedback control has been developed to estimate the velocities of a mobile robot. Among these approaches, HGO is the most popular approach used to estimate the derivative of the output signal while eliminating the disturbance effects [34,35]. Bocker and Khalil in [36] and other researchers in [37] designed full-order observers containing an extended Kalman filter observer and an extended HGO for the estimation of internal and external dynamics, respectively. These techniques were applied to the underactuated systems that can be transformed into strict feedback normal form structure. However, the key difficulties in developing an observer-based output feedback control for nonholonomic systems are that (i) the Coriolis matrix for Lagrange-based systems possesses quadratic cross terms of unmeasured velocities, (ii) nonholonomic constraints are imposed on the system, and (iii) the inability to transform into a standard strict feedback normal form. Therefore, these existing techniques cannot be directly applied to nonholonomic WMRs.

This paper presents a novel robust output feedback control approach for a class of underactuated nonholonomic systems with multi-inputs and multi-outputs (MIMO). The control technique is based on the globally defined normal form representation of the kinematic and dynamic models. Moreover, the proposed method takes into account bounded model uncertainties and external disturbances, and the absence of velocity measurements in the nonholonomic system. The controller developed in this study is applicable to both stabilization and trajectory tracking problems simultaneously.

To address the challenging task of stabilizing nonholonomic systems, a generalized normal form is introduced. This normal form representation aims to overcome the inherent difficulties associated with stabilizing such systems. By using the principles of differential geometry, it becomes feasible to change the system dynamics into a normal form through a skillfully chosen change in coordinates, known as a diffeomorphism, along with the implementation of an input–output linearization method. This transformation allows for the separation of the nonholonomic system dynamics into external and internal dynamics. The proposed normal form for uncertain nonholonomic systems integrates both kinematic and dynamic models uncertainties and external disturbance. The effect of these uncertainties is added as a perturbation term. This perturbation term enters the system through the same channel as the control input, i.e., matched uncertainty. In comparison with previous research, [1,5,7,11,12,13,14,38], the nominal values of system parameters were used for feedback linearization, and the systems were not designed to accommodate such uncertainties.

The primary effort of this research is on the stabilization problem of the normal form for the class of nonholonomic dynamic systems. This presents several challenges that need to be overcome. Firstly, the normal form representation for nonholonomic systems exhibits a nontriangular structure. Secondly, the internal dynamics of the system are non-affine in control, meaning they are highly nonlinear. Lastly, the zero dynamics of the system are not minimum phase, which further complicates the stabilization problem.

The primary contributions of this paper are as follows:

1. **Introduction of a Generalized Normal Form:** We propose a novel normal form representation for underactuated nonholonomic systems that can handle both kinematic and dynamic model uncertainties as well as external disturbances.
2. **Novel Recursive Backstepping Sliding Mode Control (BSMC):** To tackle the challenges of stabilizing nonholonomic systems, we develop a recursive backstepping SMC technique that ensures asymptotic stabilization of both external and internal dynamics. This method provides a unified solution for both trajectory tracking and stabilization problems while being robust to bounded parameter uncertainties and external disturbances.
3. **Output Feedback Control with High Gain Observer (HGO):** We design an output feedback control that employs a full-order HGO to estimate the derivative of the output function and velocity components without sensors, even in the presence of bounded uncertainties. This method achieves comparable performance to full-state feedback control.
4. **Application to Wheeled Mobile Robots:** The theoretical development is applied to the case of wheeled mobile robots, demonstrating the broad applicability of the proposed control approach to a typical benchmark problem in nonholonomic dynamic systems.

## 2. Kinematic and Dynamic Modeling for an Uncertain Nonholonomic System

Consider a class of nonholonomic dynamic system with *n* generalized coordinates $q={\left[q_{1}\ldots q_{n}\right]}^{T},q\in R^{n}$, subject to *m* nonholonomic constraints (m<n) is described as:

(1) $$J\left(q\right)\dot{q}=0$$

let $J\left(q\right)\in R^{m*n}$ be a full rank matrix linked with kinematic constraints. Suppose $[S_{1}\left(q\right)\ldots$$S_{n-m}\left(q\right)]$ are linearly independent vector fields in the null space of J(q):

(2) J(q)S(q)=0

using Equations (1) and (2) generated by the nonholonomic constraints, there exists a velocity vector $\vartheta \left(t\right)\in R^{n-m}$

(3) $$\dot{q}=S\left(q\right)\vartheta \left(t\right)$$

Moreover, the velocity vector ϑ(t) in the kinematic model (3) can be formulated in terms of systems dynamics, such as

(4) ϑ(t)=Qϖ(t)

where $Q\in R^{\left(n-m)*(n-m\right)}$ is a matrix of constant parameters, and $\varpi \left(t\right)\in R^{n-m}$ is a vector of angular wheel velocities. The kinematic model of the nonholonomic system under parameter uncertainties is derived by substituting Equation (4) into Equation (3):

(5) $$\dot{q}=C\left(q\right)\varpi \left(t\right)$$

where C(q)=S(q)Q. The dynamic model of a nonholonomic system under parameter variations and external disturbance can be expressed using the Euler–Lagrange equation:

(6) $$M\left(q\right)\ddot{q}+V\left(q,\dot{q}\right)\dot{q}+B\left(q\right)\tau_{d}=B\left(q\right)\tau -J^{T}\left(q\right)\lambda$$

where $M\left(q\right)\in R^{n*n}$ is a positive definite and symmetric inertia matrix, $B\left(q\right)\in R^{n*(n-m)}$ defines the input transformation matrix, $V\left(q,\dot{q}\right)\in R^{n*n}$ denotes the Coriolis and centripetal forces, $\tau \in R^{n-m}$ is the input torque vector, $\tau_{d}\in R^{n-m}$ represents the bounded external disturbance, and $\lambda \in R^{m}$ is constrain forces vector.

The parameter uncertainties in both kinematic and dynamic models arise due to the incorrect measurement of parameters, such as moment of inertia and mass. The actual matrix *Q*, M(q), and $V(q,\dot{q})$ can be defined as

$$\begin{array}{cc} Q & =Q_{0}+\Delta Q\\ M(q) & =M_{0}\left(q\right)+\Delta M\left(q\right)\\ V(q,\dot{q}) & =V_{0}\left(q,\dot{q}\right)+\Delta V\left(q,\dot{q}\right) \end{array}$$

where $Q_{0},M_{0}\left(q\right)$, and $V_{0}\left(q,\dot{q}\right)$ represent nominal functions, and ΔQ, ΔM(q), and $\Delta V(q,\dot{q})$ denote internal uncertainties because of parametric and nonparametric uncertainties.

**Assumption** **1.** *The model uncertainties and external disturbance are bounded by the known constants, i.e.,*

$$∥\Delta M(q)∥\leq \delta_{M},\;∥\Delta Q∥\leq \delta_{Q},\;∥\Delta V(q,\dot{q})∥\leq \delta_{V},\;∥\tau_{d}∥\leq \delta_{\tau_{d}}$$

*where $\delta_{M},\delta_{Q}$, $\delta_{V}$, and $\delta_{\tau_{d}}$ are known positive constants.*

The kinematic (5) and dynamic models (6) can be integrated to develop the state-space model for an uncertain nonholonomic system to enhance the performance of stabilizing and tracking controllers. Taking the time derivative of (5) yields

(7) $$\ddot{q}=\dot{C}\left(q\right)\varpi \left(t\right)+C\left(q\right)\dot{\varpi }\left(t\right)$$

substituting Equations (5) and (7) into Equation (6), then multiplying the resultant equation by $C^{T}$ and considering $C^{T}J^{T}\lambda =0$ because of Equation (2), one obtains

(8) $$C^{T}M\left(\dot{C}\varpi +C\dot{\varpi }\right)+C^{T}VC\varpi +C^{T}B\tau_{d}=C^{T}B\tau$$

after simplification of Equation (8) for $\dot{\varpi }$, which gives

(9) $$\dot{\varpi }=-N^{-1}\left(q\right)F\left(q,\dot{q}\right)\varpi -N^{-1}\left(q\right)\bar{B}\tau_{d}+N^{-1}\left(q\right)\bar{B}\tau$$

where $N\left(q\right)=C^{T}MC$, $F\left(q,\dot{q}\right)=C^{T}M\dot{C}+C^{T}VC$, and $\bar{B}=C^{T}B$. The state-space model is determined by combining the kinematic model (5) and dynamic Equation (9), yields

(10) $$\dot{x}=\left[\begin{array}{c} \dot{q}\\ \dot{\varpi } \end{array}\right]=\underset{f(x)}{\underset{︸}{\left[\begin{array}{c} C\varpi \\ 0 \end{array}\right]}}+\underset{p(x)}{\underset{︸}{\left[\begin{array}{c} 0\\ -N^{-1}F\varpi \end{array}\right]}}+\underset{g(x)}{\underset{︸}{\left[\begin{array}{c} 0\\ N^{-1}\bar{B} \end{array}\right]}}\tau +\underset{\delta (x,t)}{\underset{︸}{\left[\begin{array}{c} 0\\ -N^{-1}\bar{B}\tau_{d} \end{array}\right]}}$$

where $x\in R^{2n-m}$ is the state vector; $\tau \in R^{n-m}$ is the control input; and f(x), p(x), g(x), and δ(x,t) are sufficiently smooth functions in domain $D\in R^{2n-m}$. In Equation (10), *p(x)* represents the parameter uncertainties in the dynamic model, while δ(x,t) accounts for the effects of external disturbances. Table 1 describes the symbols and nomenclature used in the research paper, providing a clear reference for all variables and parameters.

**Assumption** **2.** *Assume the input transformation matrix g(x) has full rank (n−m), with g(0)≠0 to hold the controllability of the system. Furthermore, we assume that f(x) is known, while p(x), g(x), and δ(x,t) could be uncertain.*

**Remark** **1.** *The nonholonomic system (10) is strongly accessible with an accessibility rank is (2n−m). Thus, system (10) is completely controllable.*

**Remark** **2.** *The system (10) is not input-state feedback linearizable due to nonholonomic constraints. However, input–output feedback linearization can be achieved by selecting the suitable output functions [13].*

## 3. Input–Output Feedback Linearization

The input–output feedback linearization technique has been used to change the dynamics of an uncertain nonholonomic system (10) into a generalized normal form. For this purpose, a suitable set of output functions and internal dynamics variables are chosen skillfully. Then, trajectory tracking and stabilization problems can be solved using the same output functions under model uncertainties and external disturbance. Furthermore, it also provides ease in the development of HGO design for the estimation of internal dynamics and derivative of output functions. The proposed output functions can be described as

(11) $$\left[\begin{array}{c} y_{1}\\ \vdots \\ y_{n-m} \end{array}\right]=\left[\begin{array}{c} h_{1}\left(q\right)\\ \vdots \\ h_{n-m}\left(q\right) \end{array}\right]=\left[\begin{array}{c} S_{1}^{T}\left(q\right)\\ \vdots \\ S_{n-m}^{T}\left(q\right) \end{array}\right]\left[\begin{array}{c} q_{1}\\ \vdots \\ q_{n} \end{array}\right]$$

**Proposition** **1.** *Consider that the multi-input multi-output nonholonomic system (10) and (11) is partially input–output feedback linearizable. Furthermore, the largest feedback linearizable subsystem of the system (10) has 2(n−m) dimension in $x\in R^{2n-m}$ and the relative degree $\rho =\{\rho_{1},\rho_{2},\ldots ,\rho_{n-m}\}$ of each subsystem is $\rho_{i}=2$ for each output.*

**Proof.**  Taking the Lie derivative of Equation (11), yields

(12) $$\begin{array}{cc} {\dot{y}}_{i} & =L_{f}h_{i}+L_{p}h_{i}+L_{g}h_{i}\tau +L_{\delta }h_{i}\\ \; & =\Phi_{i}\left(x\right)\varpi \left(t\right),\;\;\;i=1,2,\ldots ,n-m \end{array}$$

where $\Phi_{i}\left(x\right)=\left(\partial h_{i}\left(q\right)/\partial q\right)C\left(q\right)$. Equation (12) is not explicitly dependent on the input function. Therefore, another derivative of output function (12) can be computed until input appears

(13) $$\begin{array}{cc} {\ddot{y}}_{i} & =L_{f}^{2}h_{i}+L_{p}L_{f}h_{i}+L_{g}L_{f}h_{i}\tau +L_{\delta }L_{f}h_{i}\\ \; & ={\dot{\Phi }}_{i}\left(x\right)\varpi \left(t\right)+\Phi_{i}\left(x\right)\dot{\varpi }\left(t\right) \end{array}$$

substituting $\dot{\varpi }$ from Equation (10) into Equation (13), gives

(14) $$\begin{array}{cc} {\ddot{y}}_{i} & =\alpha_{i}\left(x\right)+\Gamma_{i}\left(x\right)+\beta_{i}\left(x\right)\tau +\Psi_{i}\left(x,t\right)\\ \; & ={\dot{\Phi }}_{i}\varpi -\Phi_{i}N^{-1}F\varpi +\left(\Phi_{i}N^{-1}\bar{B}\right)\tau -\Phi_{i}N^{-1}\bar{B}\tau_{d} \end{array}$$

where

$$\begin{array}{cc} \alpha_{i}\left(x\right) & ={\dot{\Phi }}_{i}\varpi =L_{f}^{2}h_{i}\\ \Gamma_{i}\left(x\right) & =-\Phi_{i}N^{-1}F\varpi =L_{p}L_{f}h_{i}\\ \beta_{i}\left(x\right) & =\Phi_{i}N^{-1}\bar{B}=L_{g}L_{f}h_{i}\\ \Psi_{i}\left(x,t\right) & =-\Phi_{i}N^{-1}\bar{B}\tau_{d}=L_{\delta }L_{f}h_{i} \end{array}$$

The decoupling matrix β(x) is described as

$$\beta \left(x\right)=\left[\begin{array}{ccc} L_{g_{1}}L_{f}^{\rho_{1}-1}h_{1} & \ldots & L_{g_{n-m}}L_{f}^{\rho_{1}-1}h_{1}\\ L_{g_{1}}L_{f}^{\rho_{2}-1}h_{2} & \ldots & L_{g_{n-m}}L_{f}^{\rho_{2}-1}h_{2}\\ \vdots & \ddots & \vdots \\ L_{g_{1}}L_{f}^{\rho_{n-m}-1}h_{n-m} & \ldots & L_{g_{n-m}}L_{f}^{\rho_{n-m}-1}h_{n-m} \end{array}\right]$$

The decoupling matrix will be nonsingular if the regular conditions are satisfied in Equation (11). As a result, system (10) is partially input–output feedback linearizable by the proposed output. □

The relative degree of a nonholonomic system (10) is 2(n−m) in $x\in R^{2n-m}$, with each output having a relative degree of two. We require *m* more components to achieve a diffeomorphic transformation. Therefore, we define the following change in coordinates

(15) $$Z=T\left(x\right)=\left[\begin{array}{c} \psi_{1}\left(q\right)\\ \vdots \\ \psi_{m}\left(q\right)\\ h_{1}\left(q\right)\\ L_{f}h_{1}\left(x\right)\\ h_{2}\left(q\right)\\ L_{f}h_{2}\left(x\right)\\ \vdots \\ h_{n-m}\left(q\right)\\ L_{f}h_{n-m}\left(x\right) \end{array}\right]\overset{\mathrm{def}}{=}\left[\begin{array}{c} \eta_{1}\\ \vdots \\ \eta_{m}\\ \xi_{1,1}\\ \xi_{1,2}\\ \xi_{2,1}\\ \xi_{2,2}\\ \vdots \\ \xi_{n-m,1}\\ \xi_{n-m,2} \end{array}\right]$$

The variables $\eta_{1}$ to $\eta_{m}$ and $\xi_{1,1}$ to $\xi_{n-m,2}$ define the internal and external dynamics of a nonholonomic system (10), respectively. The internal dynamics η is chosen to ensure that T(x) in (15) is a legitimate diffeomorphism on a domain $D_{0}\subset D$ in $R^{\left(2n-m\right)}$ and satisfy the following requirements:

(16) $$\psi \left(0\right)=0,\;\mathrm{and}\;\frac{\partial \psi }{\partial x}g\left(x\right)=0,\;\;\;\forall \;x\in D_{0}$$

The suitable choice of internal dynamics ψ(q) that not only satisfies the conditions in (16) but also provides an ease to attain asymptotic stabilization of η dynamics, would be

(17) η=ψ(q)=J(q)q

If the Jacobian matrix $\left[\begin{array}{c} \frac{\partial T}{\partial x} \end{array}\right]$ of the map T(x) is invertible at a point $x_{0}\in D$, then Equation (15) will be a valid diffeomorphism.

$$\frac{\partial T}{\partial x}=\left[\begin{array}{ccc} \frac{\partial \eta_{1}}{\partial x_{1}} & \ldots & \frac{\partial \eta_{1}}{\partial x_{\left(2n-m\right)}}\\ \vdots & \ddots & \vdots \\ \frac{\partial \eta_{m}}{\partial x_{1}} & \ldots & \frac{\partial \eta_{m}}{\partial x_{\left(2n-m\right)}}\\ \frac{\partial \xi_{1,1}}{\partial x_{1}} & \ldots & \frac{\partial \xi_{1,1}}{\partial x_{\left(2n-m\right)}}\\ \vdots & \ddots & \vdots \\ \frac{\partial \xi_{n-m,2}}{\partial x_{1}} & \ldots & \frac{\partial \xi_{n-m,2}}{\partial x_{\left(2n-m\right)}} \end{array}\right]$$

Assuming that (∂T/∂x) has full rank for all $x\in R^{\left(2n-m\right)}$, the map T(x) is globally diffeomorphism. Therefore, one can apply the coordinates transformation (15) to change the dynamics of the system (10) into a normal form representation:

(18a)η˙=∂ψ∂qf(x)=F(η,ξ1,2)ξn−m,1(18b)ξ˙i,1=ξi,2(18c)ξ˙i,2=α¯i(η,ξ)+Γ¯i(η,ξ)+β¯i(η,ξ)τ+Ψ¯i(η,ξ,t)(18d)yi=ξi,1

where $\bar{\alpha }\left(\eta ,\xi \right)=\alpha \left(T^{-1}\left(Z\right)\right),\;\bar{\Gamma }\left(\eta ,\xi \right)=\Gamma \left(T^{-1}\left(Z\right)\right)$, and $\bar{\beta }\left(\eta ,\xi \right)=\beta \left(T^{-1}\left(Z\right)\right)$. Equations (18a)–(18d) can be rewritten in a more compact form notation

(19a)η˙=F(η,ξ1,2)ξn−m,1(19b)ξ˙=Aξ+B[α¯(η,ξ)+Γ¯(η,ξ)+β¯(η,ξ)τ+Ψ¯(η,ξ)](19c)y=Cξ

where $\xi \in R^{2(n-m)}$; $\eta \in R^{m}$; and A,B, and C can be defined as

$$A=\mathrm{block}\;\mathrm{diag}\left[A_{1},A_{2},\ldots ,A_{n-m}\right],\;\;A_{i}=\left[\begin{array}{cc} 0 & 1\\ 0 & 0 \end{array}\right]$$

$$B=\mathrm{block}\;\mathrm{diag}\left[B_{1},B_{2},\ldots ,B_{n-m}\right],\;\;B_{i}=\left[\begin{array}{c} 0\\ 1 \end{array}\right]$$

$$C=\mathrm{block}\;\mathrm{diag}\left[C_{1},C_{2},\ldots ,C_{n-m}\right],\;\;C_{i}=\left[\begin{array}{cc} 1 & 0 \end{array}\right]$$

The following nonlinear feedback control is applied to the external dynamics (19b) of the system for obtaining input–output feedback linearization and decoupling

(20) $$\tau \left(\eta ,\xi \right)={\bar{\beta }}_{0}^{-1}\left(\eta ,\xi \right)\left(u-\bar{\alpha }\left(\eta ,\xi \right)-{\bar{\Gamma }}_{0}\left(\eta ,\xi \right)\right)$$

where *u* is the auxiliary control input, and ${\bar{\beta }}_{0}\left(\eta ,\xi \right)$ and ${\bar{\Gamma }}_{0}\left(\eta ,\xi \right)$ are the nominal values of $\bar{\beta }\left(\eta ,\xi \right)$ and $\bar{\Gamma }\left(\eta ,\xi \right)$, respectively. Due to the parameter uncertainties, these values are replaced by their estimated values in the feedback control law (20). Through this process, the control action will be divided into continuous and switching components, to minimize the amplitude of the switching component to mitigate the chattering effect. After, substituting control law (20) into (19b), we obtained

(21) $$\dot{\xi }=A\xi +B\left[u+\Delta_{d}\left(\eta ,\xi ,u,t\right)\right]$$

where perturbation term $\Delta_{d}\left(\eta ,\xi ,u,t\right)$ can be defined as

$$\begin{array}{cc} \Delta_{d}\left(\eta ,\xi ,u,t\right) & =\left(\bar{\Gamma }-{\bar{\Gamma }}_{0}\right)-\left(\bar{\beta }{\bar{\beta }}_{0}^{-1}-I\right)\left(\bar{\alpha }+{\bar{\Gamma }}_{0}\right)+\bar{\Psi }+\left(\bar{\beta }{\bar{\beta }}_{0}^{-1}-I\right)u \end{array}$$

We assume that perturbation term $\Delta_{d}\left(\eta ,\xi ,u,t\right)$ satisfies the bound

(22) $$∥\Delta_{d}\left(\eta ,\xi ,u,t\right)∥⩽\sigma \left(\eta ,\xi \right)+a∥u∥$$

Thus, to satisfy Equation (22), we need the inequalities

(23a)(β¯β¯0−1−I)⩽a<1(23b)(Γ¯−Γ¯0)−(β¯β¯0−1−I)(α¯+Γ¯0)+Ψ¯⩽σ(η,ξ)

to hold over a domain that contains the origin for some continuous function σ(η,ξ). Inequality (23a) is restrictive because it puts a definite limit on the perturbation $\left(\bar{\beta }{\bar{\beta }}_{0}^{-1}-I\right)$ to achieve asymptotic stabilization of equilibrium point at the origin. Inequality (23b), conversely, is not restrictive, because we do not require σ to be small. Substituting Equation (22) into Equation (21) gives

(24) $$\dot{\xi }=A\xi +B\left[u+\sigma \left(\eta ,\xi \right)+a∥u∥\right]$$

using the results of Equation (24), we consider a generalized nontriangular normal form (19a)–(19c) for the class of nonholonomic system with (n−m) = 2 inputs that can be transformed to the following form:

(25) $$\begin{array}{cc} \Sigma_{1} & \left\{\begin{array}{c} \begin{array}{cc} {\dot{\xi }}_{1,1} & =\xi_{1,2}\\ {\dot{\xi }}_{1,2} & =u_{1}+\sigma_{1}\left(\eta ,\xi \right)+a_{1}\left|u_{1}\right| \end{array} \end{array}\right. \end{array}$$

(26) $$\begin{array}{cc} \Sigma_{2} & \left\{\begin{array}{c} \begin{array}{cc} \dot{\eta } & =F\left(\eta ,\xi_{1,2}\right)\xi_{2,1}\\ {\dot{\xi }}_{2,1} & =\xi_{2,2}\\ {\dot{\xi }}_{2,2} & =u_{2}+\sigma_{2}\left(\eta ,\xi \right)+a_{2}\left|u_{2}\right| \end{array} \end{array}\right. \end{array}$$

The generalization of obtained results to the case of (n−m) inputs is trivial. If we set $\xi_{1,2}=\xi_{2,1}=0$, the origin of η=0 is not asymptotically stable for the zero dynamics of

$$\dot{\eta }=F\left(\eta ,0\right)0$$

Thus, the zero dynamics in (26) is not minimum phase.

**Remark** **3.** *In contrast to nonlinear systems in a strict feedback form, this paper primarily focuses on the stabilization and tracking issues of nonholonomic systems represented in normal form (25) and (26), which pose the following difficulties:*

*1.*  *The proposed normal form has a nontriangular structure.*
*2.*  *Internal dynamics of the system is non-affine in control, i.e., highly nonlinear.*
*3.*  *The zero dynamics of the system is not minimum phase.*

## 4. Trajectory Tracking Controller

This section aims to develop trajectory tracking control for an uncertain nonholonomic system given in (25) and (26). The reference trajectories can be defined as

(27) $$\begin{array}{cc} {\dot{\eta }}_{r} & =F\left(\eta_{r},\xi_{1,2r}\right)\xi_{2,1r}\\ {\dot{\xi }}_{i,1r} & =\xi_{i,2r}\\ {\dot{\xi }}_{i,2r} & =u_{ir} \end{array}$$

**Assumption** **3.** *Assume $\eta_{r}$, $\xi_{i,1r}$, $\xi_{i,2r}$, and their derivatives are all bounded for all t≥0. Furthermore, the reference signals $\eta_{r}$$\xi_{i,1r}$, $\xi_{i,2r}$, and their derivatives are available on-line.*

where tracking errors are described as

(28) $$\begin{array}{cc} \eta_{e} & =\eta -\eta_{r}\\ \xi_{i,1e} & =\xi_{i,1}-\xi_{i,1r}\\ \xi_{i,2e} & =\xi_{i,2}-\xi_{i,2r} \end{array}$$

and error dynamics of trajectory tracking can be determined by computing the time derivative of (28), which gives

(29a)Σ1ξ˙1,1e=ξ1,2eξ˙1,2e=u1+σ1(η,ξ)+a1|u1|−ξ˙1,2r(29b)Σ2η˙e=M[ηe+ηr(t),ξ1,2e+ξ1,2r(t)]ξ2,1e+[M(.)−N(ηr(t),ξ1,2r(t))]ξ2,1r(t)=:F1(ξ1,2e,ξ2,1e,ηe,t)ξ˙2,1e=ξ2,2eξ˙2,2e=u2+σ2(η,ξ)+a2|u2|−ξ˙2,2r

**Step 1: Backstepping SMC for Subsystem $\Sigma_{1}$**

**Assumption** **4.** *Suppose there exists a virtual state feedback control $\xi_{1,2e}=\varphi_{1}\left(\xi_{1,1e}\right)$ with $\varphi_{1}\left(0\right)=0$; such that*

(30) $${\dot{\xi }}_{1,1e}=\xi_{1,2e}=\varphi_{1}\left(\xi_{1,1e}\right)$$

*is asymptotically stable, i.e., $\xi_{1,1e}\to zero$ as t→∞. Furthermore, there is a Lyapunov function $V_{1}\left(\xi_{1,1e}\right)$ that is positive definite and satisfies the equation*

$$\frac{\partial V_{1}}{\partial \xi_{1,1e}}\varphi_{1}\left(\xi_{1,1e}\right)\leq -k_{1}V_{1}\left(\xi_{1,1e}\right),\;\;\;k_{1}>0.$$

To design a backstepping SMC, we start by designing the sliding manifold, $s_{1}=\xi_{1,2e}-\varphi_{1}\left(\xi_{1,1e}\right)=0$ such that, when the motion is restricted to a manifold, *Assumption 4* applies. We then apply change in variables $s_{1}=\xi_{1,2e}-\varphi_{1}\left(\xi_{1,1e}\right)$ to transform the dynamics of system (29a) and (29b) into an equivalent representation

(31a)Σ1ξ˙1,1e=φ1(ξ1,1e)+s1s˙1=u1+σ1(η,ξ)+a1|u1|−ξ˙1,2r−φ˙1(ξ1,1e)(31b)Σ2η˙e=M[ηe+ηr,φ1+s1+ξ1,2r]ξ2,1e+[M(.)−N(.)]ξ2,1r(t)=:F1(ξ1,1e,ξ2,1e,s1,ηe,t)ξ˙2,1e=ξ2,2eξ˙2,2e=u2+σ2(η,ξ)+a2|u2|−ξ˙2,2r

where ${\dot{\varphi }}_{1}\left(\xi_{1,1e}\right)=\frac{\partial \varphi_{1}}{\partial \xi_{1,1e}}\left(\varphi_{1}+s_{1}\right)$. Later, $u_{1}$ will be formulated to bring $s_{1}$ to zero in finite time and remain there for all future time.

**Theorem** **1.** *Consider the subsystem (31a). Suppose Assumption 4 holds and the uncertainty satisfies the inequality (22). Let the following backstepping SMC be defined by $u_{1}$*

(32) $$u_{1}=-\frac{u_{1eq}}{\left(1-a_{1}\right)}-\gamma_{1}\left(\eta ,\xi \right)\;sat\left(\frac{s_{1}}{\mu_{1}}\right)$$

*where $\mu_{1}$ is a positive constant and*

(33) $$\begin{array}{cc} u_{1eq} & =-{\dot{\xi }}_{1,2r}-{\dot{\varphi }}_{1}+\frac{\partial V_{1}}{\partial \xi_{1,1e}}\\ \gamma_{1}\left(\eta ,\xi \right) & \geq \frac{\sigma_{1}\left(\eta ,\xi \right)}{\left(1-a_{1}\right)}+\gamma_{01},\;\;\;\;and\;\gamma_{01}>0 \end{array}$$

*Suppose $\mu_{1}$ is chosen such that the error dynamics of subsystem (31a) with arbitrary initial conditions are bounded and exponentially converge to zero for all t ≥ 0 and reach the set $\left\{\left|s_{1}\right|\leq \mu_{1}\right\}$ in finite time.*

**Proof.**  Consider the Lyapunov function

$$V_{2}\left(\xi_{1,1e},s_{1}\right)=V_{1}\left(\xi_{1,1e}\right)+\frac{1}{2}s_{1}^{2}$$

Taking ${\dot{V}}_{2}\left(\xi_{1,1e},s_{1}\right)$ along the trajectories of (31a), yields

$$\begin{array}{cc} {\dot{V}}_{2} & =\frac{\partial V_{1}}{\partial \xi_{1,1e}}\left(\varphi_{1}+s_{1}\right)+\left|s_{1}\right|\left\{u_{1}+\sigma_{1}+a_{1}\left|u_{1}\right|-{\dot{\xi }}_{1,2r}-{\dot{\varphi }}_{1}\right\}\\ \; & \leq -k_{1}V_{1}+\left|s_{1}\right|\left\{u_{1}+\sigma_{1}+a_{1}\left|u_{1}\right|-{\dot{\xi }}_{1,2r}+\frac{\partial V_{1}}{\partial \xi_{1,1e}}-{\dot{\varphi }}_{1}\right\} \end{array}$$

To reduce the chattering effect, a high-slope saturation function has been used in place of the signum function as substituting control law $u_{1}$ (32) and (33) in the above equation, gives

$$\begin{array}{cc} {\dot{V}}_{2} & \leq -k_{1}V_{1}+\left|s_{1}\right|\{\sigma_{1}\left(\eta ,\xi \right)-{\dot{\xi }}_{1,2r}-{\dot{\varphi }}_{1}+\frac{\partial V_{1}}{\partial \xi_{1,1e}}-u_{1eq}-\gamma_{1}\left(\eta ,\xi \right)\left(1-a_{1}\right)\}\\ \; & \leq -k_{1}V_{1}+\left|s_{1}\right|\left\{\sigma_{1}\left(\eta ,\xi \right)-\gamma_{1}\left(\eta ,\xi \right)\left(1-a_{1}\right)\right\}\\ \; & \leq -k_{1}V_{1}-\gamma_{01}\left(1-a_{1}\right)\left|s_{1}\right| \end{array}$$

Therefore, every trajectory reaches the manifold (surface) $s_{1}=\xi_{2e}-\varphi_{1}\left(\xi_{1,1e}\right)=0$ in finite time and cannot leave the manifold because of ${\dot{V}}_{2}\leq -k_{1}V_{1}-\gamma_{01}\left(1-a_{1}\right)\left|s_{1}\right|$. One also observes that whenever $\left|s_{1}\left(0\right)\right|>\mu_{1}$, $\left|s_{1}\left(t\right)\right|$ will reach the set $\left\{\left|s_{1}\right|\leq \mu_{1}\right\}$ in finite time and maintain inside for all future time. □

**Step 2: Backstepping SMC for Subsystem $\Sigma_{2}$**

**Assumption** **5.** *The internal dynamics $\eta_{e}$ in (31b) is asymptotically stable using the virtual state feedback control*

(34) $$\xi_{2,1e}=\varphi_{2}\left(\eta_{e}\right),\;\;with\;\varphi_{2}\left(0\right)=0$$

*Furthermore, the internal dynamics in (31b)*

$$\begin{array}{cc} {\dot{\eta }}_{e} & =M\left(.\right)\varphi_{2}\left(\eta_{e}\right)+\left[M\left(.\right)-N\left(.\right)\right]\xi_{2,1r}\left(t\right)\\ \; & =:F_{2}\left(\xi_{1,1e},s_{1},\eta_{e},t\right) \end{array}$$

*is input-to-state stable with respect to $\xi_{1,1e}$ and $s_{1}$, a positive definite Lyapunov candidate $V_{3}\left(\eta_{e}\right)$ exists that satisfies the inequality*

$$\begin{array}{cc} \; & b_{1}\left(∥\eta_{e}∥\right)\leq V_{3}\left(\eta_{e}\right)\leq b_{2}\left(∥\eta_{e}∥\right)\\ \; & \frac{\partial V_{3}}{\partial \eta_{e}}F_{2}\left(0,0,\eta_{e},t\right)\leq -k_{3}\left(∥\eta_{e}∥\right) \end{array}$$

*where $b_{1}$, $b_{2}$, and $k_{3}$ are class K functions.*

To apply the backstepping control, a change in coordinates $z=\xi_{2,1e}-\varphi_{2}\left(\eta_{e}\right)$ will transform the dynamics of (31b) into equivalent representation

(35) $$\begin{array}{cc} \Sigma_{2} & \left\{\begin{array}{c} \begin{array}{cc} {\dot{\eta }}_{e} & =M\left(.\right)\left[\varphi_{2}+z\right]+\left[M\left(.\right)-N\left(.\right)\right]\xi_{2,1r}\\ \; & =:F_{2}\left(\xi_{1,1e},s_{1},\eta_{e},t\right)+M\left(.\right)z\\ \dot{z} & =\xi_{2,2e}-{\dot{\varphi }}_{2}\left(\eta_{e}\right)\\ {\dot{\xi }}_{2,2e} & =u_{2}+\sigma_{2}\left(\eta ,\xi \right)+a_{2}\left|u_{2}\right|-{\dot{\xi }}_{2,2r} \end{array} \end{array}\right. \end{array}$$

where ${\dot{\varphi }}_{2}\left(\eta_{e}\right)=\frac{\partial \varphi_{2}}{\partial \eta_{e}}\left[F_{2}\left(\xi_{1,1e},s_{1},\eta_{e},t\right)+M\left(.\right)z\right]$

**Proposition** **2.** *Consider the subsystem (35). Let the Assumptions 4 and 5 hold. Then, there exists a virtual state feedback control*

(36) $$\xi_{2,2e}=\varphi_{3}\left(\eta_{e},z\right)=-\frac{\partial V_{3}}{\partial \eta_{e}}M\left(.\right)+{\dot{\varphi }}_{2}\left(\eta_{e}\right)-k_{4}z$$

*with $\varphi_{3}\left(0,0\right)=0,$ that asymptotically stabilizes the error dynamics of $\eta_{e}$ and z in subsystem (35), where $k_{4}>0$.*

**Proof.**  Consider the Lyapunov function

$$V_{4}\left(\eta_{e},z\right)=V_{3}\left(\eta_{e}\right)+\frac{1}{2}z^{2}$$

Taking ${\dot{V}}_{4}$ along the trajectories of subsystem (35)

$$\begin{array}{cc} {\dot{V}}_{4}\left(\eta_{e},z\right) & =\frac{\partial V_{3}}{\partial \eta_{e}}\left[F_{2}\left(0,0,\eta_{e},t\right)+M\left(.\right)z\right]+z\left(\xi_{2,2e}-{\dot{\varphi }}_{2}\left(\eta_{e}\right)\right)\\ \; & \leq \frac{\partial V_{3}}{\partial \eta_{e}}F_{2}\left(\eta_{e},t\right)+z[\xi_{2,2e}+\frac{\partial V_{3}}{\partial \eta_{e}}M\left(.\right)-{\dot{\varphi }}_{2}\left(\eta_{e}\right)] \end{array}$$

and substituting virtual feedback control (36) in the above equation

$${\dot{V}}_{4}\left(\eta_{e},z\right)=-k_{3}\left(∥\eta_{e}∥\right)-k_{4}z^{2}$$

Thus, ${\dot{V}}_{4}\left(\eta_{e},z\right)$ is negative definite. Therefore, $\eta_{e}$ and *z* will remain bounded and converge to the origin as t→∞. □

To design a robust backstepping SMC for the entire closed-loop subsystem (35), we start by designing the sliding manifold (surface) $s_{2}=\xi_{2,2e}-\varphi_{3}\left(\eta_{e},z\right)=0$ such that, when the motion is restricted to manifold, Proposition 2 applies. We then apply change in variables $s_{2}=\xi_{2,2e}-\varphi_{3}\left(\eta_{e},z\right)$ that transforms the dynamics of subsystem (35) into an equivalent representation

(37) $$\begin{array}{cc} \Sigma_{2} & \left\{\begin{array}{c} \begin{array}{cc} {\dot{\eta }}_{e} & =F_{2}\left(\xi_{1,1e},s_{1},\eta_{e},t\right)+M\left(.\right)z\\ \dot{z} & =-\frac{\partial V_{3}}{\partial \eta_{e}}M\left(.\right)-k_{4}z+s_{2}\\ {\dot{s}}_{2} & =u_{2}+\sigma_{2}\left(\eta ,\xi \right)+a_{2}\left|u_{2}\right|-{\dot{\xi }}_{2,2r}-{\dot{\varphi }}_{3}\left(\eta_{e},z\right) \end{array} \end{array}\right. \end{array}$$

where ${\dot{\varphi }}_{3}\left(\eta_{e},z\right)=\frac{\partial \varphi_{3}}{\partial \eta_{e}}{\dot{\eta }}_{e}+\frac{\partial \varphi_{3}}{\partial z}\dot{z}$. Now, $u_{2}$ will be developed to bring $s_{2}$ to zero in finite time and remain there for all future time.

**Theorem** **2.** *Consider the closed-loop subsystem (37). Suppose Proposition 2 is satisfied and the uncertainty meets the inequality (22). Let the following backstepping SMC be defined by $u_{2}$*

(38) $$u_{2}=-\frac{u_{2eq}}{\left(1-a_{2}\right)}-\gamma_{2}\left(\eta ,\xi \right)\;sat\left(\frac{s_{2}}{\mu_{2}}\right)$$

*where $\mu_{2}$ is a positive constant and*

(39) $$\begin{array}{cc} u_{2eq} & =-{\dot{\xi }}_{2,2r}-{\dot{\varphi }}_{3}\left(\eta_{e},z\right)+\frac{\partial V_{4}}{\partial z}\\ \gamma_{2}\left(\eta ,\xi \right) & \geq \frac{\sigma_{2}\left(\eta ,\xi \right)}{\left(1-a_{2}\right)}+\gamma_{02},\;\;\;\;\;and\;\gamma_{02}>0 \end{array}$$

*Furthermore, suppose $\mu_{2}$ is chosen such that the error dynamics of subsystem (37) with arbitrary initial conditions are bounded and exponentially converge to the origin *∀* t ≥ 0 and reach the set $\left\{\left|s_{2}\right|\leq \mu_{2}\right\}$ in finite time.*

**Proof.**  Consider the composite Lyapunov function

$$V_{5}\left(\eta_{e},z,s_{2}\right)=V_{4}+\frac{1}{2}s_{2}^{2}$$

Taking ${\dot{V}}_{5}$, we obtained

(40) $$\begin{array}{cc} {\dot{V}}_{5} & \leq -k_{3}\left(∥\eta_{e}∥\right)-k_{4}z^{2}+\left|s_{2}\right|\{u_{2}+\sigma_{2}\left(\eta ,\xi \right)+a_{2}\left|u_{2}\right|-{\dot{\xi }}_{2,2r}+\frac{\partial V_{4}}{\partial z}-{\dot{\varphi }}_{3}\left(\eta_{e},z\right)\} \end{array}$$

and substituting control law $u_{2}$ (38) in above equation, yields

$$\begin{array}{cc} {\dot{V}}_{5} & \leq -k_{3}\left(∥\eta_{e}∥\right)-k_{4}z^{2}+\left|s_{2}\right|\{\sigma_{2}\left(\eta ,\xi \right)-{\dot{\xi }}_{2,2r}-{\dot{\varphi }}_{3}\left(\eta_{e},z\right)+\frac{\partial V_{4}}{\partial z}-u_{2eq}-\gamma_{2}\left(\eta ,\xi \right)\left(1-a_{2}\right)\}\\ \; & \leq -k_{3}\left(∥\eta_{e}∥\right)-k_{4}z^{2}+\left|s_{2}\right|\left\{\sigma_{2}\left(\eta ,\xi \right)-\gamma_{2}\left(\eta ,\xi \right)\left(1-a_{2}\right)\right\}\\ \; & \leq -k_{3}\left(∥\eta_{e}∥\right)-k_{4}z^{2}-\gamma_{02}\left(1-a_{2}\right)\left|s_{2}\right| \end{array}$$

Because ${\dot{V}}_{5}\leq -k_{3}\left(.\right)-k_{4}\left(.\right)-\gamma_{02}\left(1-a_{2}\right)\left|s_{2}\right|$, every trajectory reaches the manifold $s_{2}=\xi_{2,2e}-\varphi_{3}\left(\eta_{e},z\right)=0$ in finite time and can not leave the manifold. One also observes that whenever $\left|s_{2}\left(0\right)\right|>\mu_{2}$, $\left|s_{2}\left(t\right)\right|$ will reach the set $\left\{\left|s_{2}\right|\leq \mu_{2}\right\}$ in finite time and maintain inside thereafter. □

## 5. Backstepping SMC for Stabilization

Consider the nonholonomic system (25) and (26). After applying the same virtual state feedback control developed in (30), (34), and (36) and the change in coordinates defined in previous Section 4 (with all reference signals set to zero), we can rewrite subsystems (31a) and (37) for the stabilization of the nonholonomic system as

(41a)Σ1ξ˙1,1=φ1(ξ1,1)+s1s1˙=u1+σ1(η,ξ)+a1|u1|−φ˙1(ξ1,1)(41b)Σ2η˙=F2(ξ1,1,s1,η)+M(η,φ1(ξ1,1)+s)zz˙=−∂V3∂ηM(.)−k4z+s2s˙2=u2+σ2(η,ξ)+a2|u2|−φ˙3(η,z)

**Theorem** **3.** *Consider the system (41a) and (41b), where the uncertainty term satisfies the inequality (22). Then, the robust backstepping SMC $u_{1}$ (32) and (33) and $u_{2}$ (38) and (39) (with all reference signals set to zero), yields asymptotic stabilization to the origin and reach the set $\left\{\left|s_{i}\right|\leq \mu_{i}\right\}$ in finite time.*

## 6. Output Feedback Control

Suppose the uncertain nonholonomic system (18a)–(18d). Consider an internal observer comprises of internal dynamics η and a virtual output ζ for the auxiliary system

(42) $$\begin{array}{cc} \dot{\eta } & =F\left(\eta ,\xi_{1,2}\right)\xi_{n-m,1} \end{array}$$

(43) $$\begin{array}{cc} \zeta & =\eta \end{array}$$

**Remark** **4.** *η=ψ(q) dynamics is a function of generalized coordinates $q={\left[q_{1}\ldots q_{n}\right]}^{T}$ as described in (15). Assuming that output functions $y_{i}=h_{i}\left(q\right)$ in Equation (11) are selected to ensure that all generalized coordinates q can be measured through the output functions.*

Thus, the internal observer can be formulated as

(44) $$\dot{\hat{\eta }}=F\left(\hat{\eta },{\hat{\xi }}_{1,2}\right){\hat{\xi }}_{n-m,1}+L_{\eta }\left(\zeta -\hat{\eta }\right)$$

where $L_{\eta }$ is the observer gain. The high gain observer for the estimation of external dynamics of the nonholonomic system (18b) and (18c) is given by

(45) $$\begin{array}{cc} {\dot{\hat{\xi }}}_{i,1} & ={\hat{\xi }}_{i,2}+\left(h_{i,1}/\epsilon_{i}\right)\left(y_{i}-{\hat{\xi }}_{i,1}\right)\\ {\dot{\hat{\xi }}}_{i,2} & ={\bar{\alpha }}_{i}\left(\hat{\eta },\hat{\xi }\right)+{\bar{\Gamma }}_{0i}\left(\hat{\eta },\hat{\xi }\right)+{\bar{\beta }}_{0i}\left(\hat{\eta },\hat{\xi }\right)\tau +\left(h_{i,2}/\epsilon_{i}^{2}\right)\left(y_{i}-{\hat{\xi }}_{i,1}\right) \end{array}$$

The observer gains $h_{i}^{*}={\left[h_{i,1}/\epsilon_{i},h_{i,2}/\epsilon_{i}^{2}\right]}^{T}$ and the gain parameters $h_{i,1},h_{i,2}$ are selected such that the polynomial $s^{2}+h_{i,1}s+h_{i,2}$ is Hurwitz. Furthermore, the parameter $\epsilon_{i}$ is a small positive constant.

The closed-loop system (19a)–(19c) under output feedback control is written as

(46a)η˙=F(η,ξ1,2)ξn−m,1(46b)ξ˙=Aξ+B[α¯(η,ξ)+Γ¯(η,ξ)+β¯(η,ξ)τ(η^,ξ^)+Ψ¯(η,ξ)]

where the output feedback control is designed as

(47a)τ(η^,ξ^)=β¯0−1(η^,ξ^)[u−α¯(η^,ξ^)−Γ¯0(η^,ξ^)](47b)ui=−uieq(η^,ξ^)(1−ai)−γi(η^,ξ^)sat(s^iμi)

where $\hat{\eta }$ and $\hat{\xi }$ are estimated by the full-order HGO that integrates the internal observer (44) with the HGO (45) written in a compact notation

(48a)η^˙=F(η^,ξ^1,2)ξ^n−m,1+Lη(ζ−η^)(48b)ξ^˙=Aξ^+B[α¯(η^,ξ^)+Γ¯0(η^,ξ^)+β¯0(η^,ξ^)τ(η^,ξ^)]+Hξ(y−Cξ^)

where $H_{\xi }=\mathrm{block}\;\mathrm{diag}\left[H_{1},H_{2},\ldots ,H_{n-m}\right],\;\;H_{i}=\left[\begin{array}{c} h_{i,1}/\epsilon_{i}\\ h_{i,2}/\epsilon_{i}^{2} \end{array}\right]$

Consider the scaled estimation error

(49) $$\begin{array}{cc} \tilde{\eta } & =\eta -\hat{\eta } \end{array}$$

(50) $$\begin{array}{cc} \chi_{i,j} & =\left(\xi_{i,j}-{\hat{\xi }}_{i,j}\right)/\epsilon_{i}^{\rho -j},\;\;\;1\leq j\leq \rho \end{array}$$

Let,

$$\begin{array}{cc} \chi & ={\left[\chi_{1,1},\chi_{1,2},\ldots ,\chi_{n-m,1},\chi_{n-m,2}\right]}^{T},\\ D(\epsilon ) & =\mathrm{block}\;\mathrm{diag}[D_{1},D_{2}\ldots ,D_{n-m}],\\ D_{i}\left(\epsilon \right) & =\mathrm{diag}[\epsilon_{i}^{\rho -1},\ldots ,1] \end{array}$$

Accordingly, we can write D(ϵ)χ = $\xi -\hat{\xi }$. The dynamics of estimation error can be written as

(51) $$\begin{array}{c} \begin{array}{cc} \dot{\tilde{\eta }} & =F\left(\hat{\eta }+\tilde{\eta },{\hat{\xi }}_{1,2}+\chi_{1,2}\right)\left({\hat{\xi }}_{2,1}+\epsilon_{2}\chi_{2,1}\right)-F\left(\hat{\eta },{\hat{\xi }}_{1,2}\right){\hat{\xi }}_{2,1}-L_{\eta }\left(\zeta -\hat{\eta }\right)\\ & ≜f_{\eta }\left(\hat{\eta },\hat{\xi },\tilde{\eta },D\left(\epsilon \right)\chi ,t\right) \end{array} \end{array}$$

(52) $$\begin{array}{cc} \epsilon \dot{\chi } & =\Lambda \chi +\epsilon B[\Delta \alpha +\Delta \Gamma +\Delta \beta +\bar{\Psi }\left(\hat{\eta }+\tilde{\eta },\hat{\xi }+D\chi \right)] \end{array}$$

where

$$\Lambda =\mathrm{block}\;\mathrm{diag}\left[\Lambda_{1},\Lambda_{2},\ldots ,\Lambda_{n-m}\right],\;\;\Lambda_{i}=\left[\begin{array}{cc} -h_{i,1} & 1\\ -h_{i,2} & 0 \end{array}\right]$$

and

$$\begin{array}{cc} \Delta \alpha & =\bar{\alpha }\left(\tilde{\eta }+\hat{\eta },\hat{\xi }+D\chi \right)-\bar{\alpha }\left(\hat{\eta },\hat{\xi }\right)\\ \Delta \Gamma & =\bar{\Gamma }\left(\tilde{\eta }+\hat{\eta },\hat{\xi }+D\chi \right)-{\bar{\Gamma }}_{0}\left(\hat{\eta },\hat{\xi }\right)\\ \Delta \beta & =\bar{\beta }\left(\tilde{\eta }+\hat{\eta },\hat{\xi }+D\chi \right)\tau -{\bar{\beta }}_{0}\left(\hat{\eta },\hat{\xi }\right)\tau \end{array}$$

The system (51) and (52) satisfies the singular perturbation form. It can be observed that decreasing ϵ diminishes the effect of uncertainties in Equation (52). Since the closed-loop system (19a)–(19c) is asymptotically stable around the origin, assume R is the region of attraction. The initial states (ξ(0),η(0))∈S, where S is any compact set in the interior of R, and $\hat{\eta }\left(0\right)\in Q_{1}$, and $\hat{\xi }\left(0\right)\in Q_{2}$, where $Q_{1}$ and $Q_{2}$ are any compact subsets of $R^{m}$ and $R^{\rho (n-m)}$, respectively. Therefore, $\tilde{\eta }\left(0\right)=\eta \left(0\right)-\hat{\eta }\left(0\right)$, and $\chi \left(0\right)=D^{-1}\left(\epsilon \right)\left[\xi \left(0\right)-\hat{\xi }\left(0\right)\right]$. Furthermore, trajectories starting in $\left(\hat{\eta }\left(0\right),\hat{\xi }\left(0\right)\right)\in Q_{1}\times Q_{2}$ enter the positively invariant set W in finite time.

**Theorem** **4.** *Consider the closed-loop system (46a) and (46b) under output feedback controller (47a) and (47b), designed using the full order HGO (48a) and (48b). Theorems 1–3 are satisfied for the asymptotic tracking and stabilization of the closed-loop system (19), respectively, and assume R is its region of attraction. Let Q be any compact subset of $R^{\rho (n-m)+m}$, and S be any compact set in the interior of R. Then, $\mu_{i}^{*}>0$ and $\epsilon_{i}^{*}>0$ exist such that for every $0<\mu_{i}<\mu_{i}^{*}$ and $0<\epsilon_{i}<\epsilon_{i}^{*}$, the solutions of the closed-loop systems (46a)–(46b) and (48a)–(48b), starting in S×Q, are bounded and the estimation error approaches zero as t→∞.*

**Proof.**  Consider the Lyapunov function for the estimation error of internal dynamics (51), such as

$$V_{\tilde{\eta }}\left(t,\tilde{\eta }\right)={\tilde{\eta }}^{T}P^{-1}\tilde{\eta }$$

satisfying the inequality $0<c_{1}I_{m}\leq P^{-1}\leq c_{2}I_{m}$, where $c_{1},c_{2}$ are positive constants, and $P^{-1}$ is a positive definite matrix. It also verifies that

(53) $$\begin{array}{cc} c_{1}{∥\tilde{\eta }∥}^{2} & \leq V_{\tilde{\eta }}\left(t,\tilde{\eta }\right)\leq c_{2}{∥\tilde{\eta }∥}^{2} \end{array}$$

(54) $$\begin{array}{cc} \frac{\partial V_{\tilde{\eta }}}{\partial \tilde{\eta }}f_{\eta }\left(\hat{\eta },\hat{\xi },\tilde{\eta },0,t\right) & \leq -c_{3}{∥\tilde{\eta }∥}^{2},\;\forall ∥\tilde{\eta }∥\leq c_{4},\;\forall \;t\geq t_{0} \end{array}$$

where $c_{3},c_{4}>0$ and are independent of ϵ. The compact set $Q_{1}\subset R^{m}$ will ensure that inequality (53) and (54) are valid.

Now, applying the change in coordinate Υ=t/ϵ in (52) to obtain the boundary layer model, then substituting ϵ=0, yields

(55) $$\frac{d\chi }{d\Upsilon }=\Lambda \chi$$

The Lyapunov function candidate for the boundary layer system (55) can be defined as

$$V_{\chi }\left(\chi \right)=\chi^{T}W\chi$$

where *W* is the positive definite solution of Lyapunov function $W\Lambda +\Lambda^{T}W=-I$, satisfies

$$\begin{array}{cc} \lambda_{min}\left(W\right){∥\chi ∥}^{2} & \leq V_{\chi }\left(\chi \right)\leq \lambda_{max}\left(W\right){∥\chi ∥}^{2}\\ \frac{\partial V_{\chi }}{\partial \chi }\Lambda \chi & \leq -{∥\chi ∥}^{2} \end{array}$$

Additionally, it can be shown that time derivative of $V_{\tilde{\eta }}$ and $V_{\chi }$ also satisfy

(56) $$\begin{array}{cc} {\dot{V}}_{\tilde{\eta }} & =-c_{3}{∥\tilde{\eta }∥}^{2}+k_{5}∥\chi ∥\\ {\dot{V}}_{\chi } & =-\frac{1}{\epsilon }{∥\chi ∥}^{2}+k_{6}∥\chi ∥∥W∥ \end{array}$$

where $k_{5}$ and $k_{6}$ are positive constants independent of ϵ. Therefore, for all $0<\epsilon_{i}<\epsilon_{i}^{*}$, $t\geq t_{0}$, and trajectories $(\hat{\eta }\left(0\right)$ and $\hat{\xi }\left(0\right))$ starting in $Q_{1}\times Q_{2}$, the dynamics of estimation errors $\left(\tilde{\eta },\chi \right)$ converge to origin as t→∞. □

## 7. Design Example: Differential Drive WMR

### 7.1. Problem Formulation

**Figure 1:**
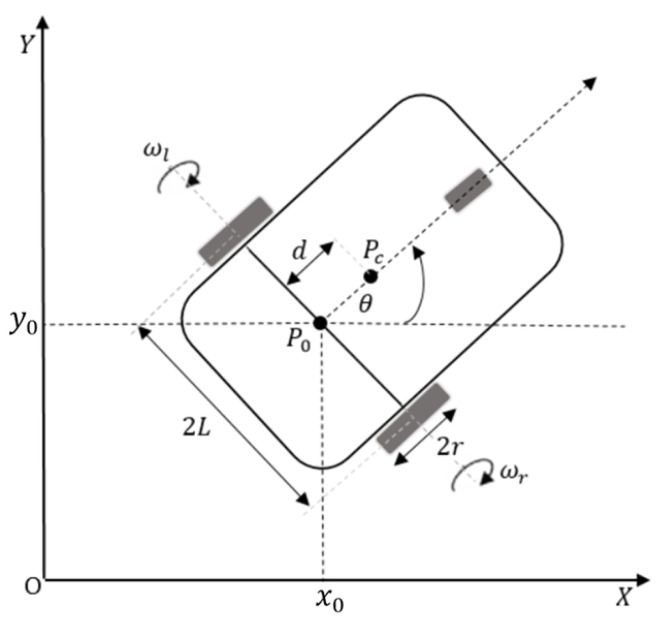
Wheeled mobile robot (WMR).

This paper presents the differential drive type (2,0) WMR as an example of performing robust output feedback stabilization and trajectory tracking, as illustrated in Figure 1.

The posture of a mobile robot in an inertial Cartesian frame O,X,Y can be described by $q={\left[x_{0},y_{0},\theta \right]}^{T}$. The parameter *r* denotes the radius of each driving wheel, separated by a distance 2L. The constraint vector J(q), S(q), and velocity vector ϑ(t) as defined in Equations (1)–(3) are described as

$$J\left(q\right)=\left[\sin\theta \;-\cos\theta \;0\right],\;S\left(q\right)=\left[\begin{array}{cc} \cos\theta & 0\\ \sin\theta & 0\\ 0 & 1 \end{array}\right],\;\vartheta =\left[\begin{array}{c} \vartheta_{1}\left(t\right)\\ \vartheta_{2}\left(t\right) \end{array}\right]$$

where $\vartheta_{1}\left(t\right)$ and $\vartheta_{2}\left(t\right)$ are the linear and angular velocities of WMR, respectively. Similarly, transformation matrix *Q* and angular wheel velocity vector ϖ from Equation (4) are defined as

$$Q=\frac{1}{2}\left[\begin{array}{cc} r & r\\ \frac{r}{L} & -\frac{r}{L} \end{array}\right],\;\varpi =\left[\begin{array}{c} \varpi_{r}\\ \varpi_{l} \end{array}\right]$$

The kinematic model of WMR by Equation (5)

(57) $$\dot{q}=\left[\begin{array}{c} {\dot{x}}_{0}\\ {\dot{y}}_{0}\\ \dot{\theta } \end{array}\right]=\left[\begin{array}{cc} p_{1}\cos\theta & p_{1}\cos\theta \\ p_{1}\sin\theta & p_{1}\sin\theta \\ p_{2} & -p_{2} \end{array}\right]\left[\begin{array}{c} \varpi_{r}\\ \varpi_{l} \end{array}\right]$$

where $p_{1}=\frac{r}{2}$ and $p_{2}=\frac{r}{2L}$. The following matrices define the dynamic model as introduced in Equation (6)

$$\begin{array}{c} M\left(q\right)=\left[\begin{array}{ccc} \bar{m} & 0 & \bar{m}d\sin\theta \\ 0 & \bar{m} & -\bar{m}d\cos\theta \\ \bar{m}d\sin\theta & -\bar{m}d\cos\theta & I \end{array}\right],\;\tau =\left[\begin{array}{c} \tau_{r}\\ \tau_{l} \end{array}\right],\\ V\left(q,\dot{q}\right)=\left[\begin{array}{c} \bar{m}d\dot{\theta }\cos\theta \\ \bar{m}d\dot{\theta }\sin\theta \\ 0 \end{array}\right],\;B\left(q\right)=\frac{1}{r}\left[\begin{array}{cc} \cos\theta & \cos\theta \\ \sin\theta & \sin\theta \\ L & -L \end{array}\right] \end{array}$$

where $\bar{m}={\bar{m}}_{c}+2{\bar{m}}_{w}$ and $I=I_{c}+2I_{w}+{\bar{m}}_{c}d^{2}+2{\bar{m}}_{w}\left(L^{2}+d^{2}\right)$. The parameters ${\bar{m}}_{w}$ and ${\bar{m}}_{c}$ are the mass of driving wheels, including rotors of the DC motor, and the mass of the mobile robot platform, respectively. The parameter $I_{w}$ represents the moment of inertia of each wheel with a motor rotor, and $I_{c}$ is the moment of inertia of the WMR platform. The output vector (11) for (n−m=2) outputs is selected as

(58) $$\left[\begin{array}{c} y_{1}\\ y_{2} \end{array}\right]=\left[\begin{array}{c} \theta \\ x_{0}\cos\theta +y_{0}\sin\theta \end{array}\right]$$

and the suitable choice of internal dynamic (17) would be

(59) $$\eta =x_{0}\sin\theta -y_{0}\cos\theta$$

where the decoupling matrix Φ(η,ξ) (12) can be defined as

$$\Phi \left(\eta ,\xi \right)=\left[\begin{array}{cc} p_{2} & -p_{2}\\ p_{1}-p_{2}\eta & p_{1}+p_{2}\eta \end{array}\right]$$

The proposed nontriangular normal form for an uncertain wheeled mobile robot, according to the notations introduced in Equations (15)–(19), we obtained

(60a)η˙=ξ1,2ξ2,1(60b)ξ˙=Aξ+B[α¯(η,ξ)+Γ¯(η,ξ)+β¯(η,ξ)τ+Ψ¯(η,ξ)](60c)y=Cξ

where

$$\begin{array}{c} \bar{\alpha }\left(\eta ,\xi \right)=\left[\begin{array}{c} 0\\ -\xi_{1,2}^{2}\xi_{2,1} \end{array}\right],\;\;\bar{\beta }\left(\eta ,\xi \right)=\left[\begin{array}{cc} P_{3} & -P_{3}\\ P_{4}-P_{3}\eta & P_{4}+P_{3}\eta \end{array}\right],\\ \bar{\Gamma }\left(\eta ,\xi \right)=\left[\begin{array}{c} P_{5}\xi_{1,2}\left(\xi_{1,2}\eta +\xi_{2,2}\right)\\ -d\xi_{1,2}^{2}-P_{5}\xi_{1,2}\eta \left(\xi_{1,2}\eta +\xi_{2,2}\right) \end{array}\right] \end{array}$$

where $P_{3}=\frac{L}{rI}$, $P_{4}=\frac{1}{r\bar{m}}$, and $P_{5}=\frac{\bar{m}d}{I}$, are calculated from the physical parameters of WMR. The mobile robot parameters such as mass ($\bar{m}$), wheel radius (*r*), distance between two wheels (2L), and moment of inertia (*I*) are assumed to be uncertain. Therefore, $P_{1},P_{2},P_{3},P_{4},$ and $P_{5}$ are also uncertain.

### 7.2. Trajectory Tracking of a WMR

Applying the nonlinear feedback control (20) to obtain input–output feedback linearization and decoupling of the WMR described in Equations (60a)–(60c), and then using the results of Equations (21)–(28), the error dynamical model (29) for trajectory tracking can be described as follows:

(61a)Σ1ξ˙1,1e=ξ1,2eξ˙1,2e=u1+σ1(η,ξ)+a1|u1|−ξ˙1,2r(61b)Σ2η˙e=(ξ1,2e+ξ1,2r(t))ξ2,1e+ξ1,2eξ2,1r(t)ξ˙2,1e=ξ2,2eξ˙2,2e=u2+σ2(η,ξ)+a2|u2|−ξ˙2,2r

The virtual control inputs (30), (34), and (36) are chosen as

(62a)ξ1,2e=φ1(ξ1,1e)=−k1ξ1,1e(62b)ξ2,1e=φ2(ηe)=−k3ηe(ξ1,2r−k1ξ1,1e)(62c)ξ2,2e=φ3(ηe,z)=−[k3/(ξ1,2r−k1ξ1,1e)]η˙e−ηeξ1,2r−k4z

Then, apply the change in variables

(63a)s1=ξ1,2e+k1ξ1,1e(63b)z=ξ2,1e+k3ηe(ξ1,2r−k1ξ1,1e)(63c)s2=ξ2,2e+[k3/(ξ1,2r−k1ξ1,1e)]η˙e+ηeξ1,2r+k4z

to transform the error dynamics of a mobile robot from Equations (61a) and (61b) into a similar form as described in Equations (31a) and (37), respectively,

(64a)Σ1ξ˙1,1e=−k1ξ1,1e+s1s˙1=u1+σ1(η,ξ)+a1|u1|−ξ˙1,2r+k1s1−k12ξ1,1e(64b)Σ2η˙e=−k3ηe−k1(z+ξ2,1r)ξ1,1e+ξ1,2rz+(z+φ2+ξ2,1r)s1z˙=−k4z−ηeξ1,2r+s2s˙2=u2+σ2(η,ξ)+a2|u2|−ξ˙2,2r−[(k32/(ξ1,2r−k1ξ1,1e))−ξ1,2r]η˙e+k4z˙+[(s1−k1ξ1,1e+ξ1,2r)/(ξ1,2r−k1ξ1,1e)]z˙

The internal dynamics $\eta_{e}$ in (64b) will remain bounded and exponentially converges to zero if the following requirement is fulfilled to evade singularity: $\left|\xi_{1,2r}\left(t\right)+\varphi_{1}\left(\xi_{1,1e}\right)\right|$ = 0, as stated in Equation (62b),

$$C1:\;\;|\xi_{1,2r}\left(t\right)|>|\varphi_{1}\left(\xi_{1,1e}\left(t\right)\right)|,\;\;\forall \;t⩾0$$

To meet the above requirement labeled **C1**, it is essential to initialize the reference trajectory $\xi_{1,2r}\left(0\right)$ and the error trajectory $\xi_{1,1e}\left(0\right)$ appropriately. Meeting the condition of **C1** means ensuring that $|\xi_{1,2r}\left(0\right)|>|\varphi_{1}\left(\xi_{1,1e}\left(0\right)\right)|$, will protect against singularity, when error dynamics $\xi_{1,1e}$ approaches zero in the initial transient phase.

We can apply the backstepping sliding mode control law $u_{1}$ (32) and (33) and $u_{2}$ (38) and (39) to the error dynamical model Equations (64a) and (64b) to perform trajectory tracking of a WMR. The values of $u_{1eq}$ and $u_{2eq}$ in (33) and (39), respectively, can be formulated as

(65) $$\begin{array}{cc} u_{1eq} & =-{\dot{\xi }}_{1,2r}-k_{1}^{2}\xi_{1,1e}+k_{1}s_{1}+\xi_{1,1e} \end{array}$$

(66) $$\begin{array}{c} \begin{array}{cc} u_{2eq} & =-{\dot{\xi }}_{2,2r}-\left[\left(k_{3}^{2}/\left(\xi_{1,2r}-k_{1}\xi_{1,1e}\right)\right)-\xi_{1,2r}\right]{\dot{\eta }}_{e}+z+[\left(s_{1}-k_{1}\xi_{1,1e}+\xi_{1,2r}\right)/\left(\xi_{1,2r}-k_{1}\xi_{1,1e}\right)\\ \; & \;\;\;+k_{4}]\dot{z} \end{array} \end{array}$$

### 7.3. Posture Stabilization of a WMR

Consider the system (61a) and (61b) with all reference signals set to zero, we obtained

(67a)Σ1ξ˙1,1=ξ1,2ξ˙1,2=u1+σ1(η,ξ)+a1|u1|(67b)Σ2η˙=ξ1,2ξ2,1ξ˙2,1=ξ2,2ξ˙2,2=u2+σ2(η,ξ)+a2|u2|

Then, employing the same virtual state feedback control developed in Equations (62a)–(62c), with all reference signals set to zero

(68a)ξ1,2=φ1(ξ1,1)=−k1ξ1,1(68b)ξ2,1=φ2(η)=k3ηk1ξ1,1(68c)ξ2,2=φ3(η,z)=(k3/k1ξ1,1e)η˙e−k4z

Later, using the results of Equations (63a) and (64b) (setting all reference signals to zero) to transform the dynamics of the system (67a) and (67b) into equivalent representation

(69a)Σ1ξ˙1,1=−k1ξ1,1+s1s1˙=u1+σ1(η,ξ)+a1|u1|−k12ξ1,1+k1s1(69b)Σ2η˙=−k3η−k1ξ1,1z+(z+φ2)s1z˙=−k4z+s2s˙2=u2+σ2(η,ξ)+a2|u2|+(k32/k1ξ1,1)η˙e−[(s1−k1ξ1,1)/(k1ξ1,1)−k4]z˙

**Proposition** **3.** *The internal dynamics η in (69b) will remain bounded and exponentially converge to zero if the given requirements are fulfilled to achieve the bounded solution of Equation (68b)*

$$\begin{array}{cc} C2: & \;K_{2}^{2}-4K_{1}>0\\ C3: & \;|\xi_{1}\left(t\right)|\neq 0\;\;\forall \;t⩾0 \end{array}$$

*where $K_{1}=\left[1+\frac{k_{1}\left(1-a_{1}\right)\gamma_{01}}{\mu_{1}}\right]$, and $K_{2}=\left[k_{1}+\frac{\left(1-a_{1}\right)\gamma_{01}}{\mu_{1}}\right]$.*

**Proof.**  In this Proposition, we will prove that Equation (68b) will remain bounded as the solution of $\xi_{1,1}\left(t\right)$ converges to the origin, ∀t⩾0. For this purpose, let us derive the solution of subsystem (69a) by substituting the control law $u_{1}$ as defined in the previous section (setting all reference signals to zero)

(70) $$\begin{array}{c} \begin{array}{cc} {\dot{\xi }}_{1,1} & =\xi_{1,2}\\ {\dot{\xi }}_{1,2} & =-K_{1}\xi_{1,1}-K_{2}\xi_{1,2} \end{array} \end{array}$$

Integrating the closed-loop system (70), we obtain

$$\left[\begin{array}{c} \xi_{1,1}\left(t\right)\\ \xi_{1,2}\left(t\right) \end{array}\right]=e^{At}\left[\begin{array}{c} \xi_{1,1}\left(0\right)\\ \xi_{1,2}\left(0\right) \end{array}\right]$$

where

$$e^{At}=\left[\begin{array}{cc} b_{1}e^{\lambda_{1}\left(t\right)}-b_{2}e^{\lambda_{2}\left(t\right)} & b_{3}e^{\lambda_{1}\left(t\right)}-b_{3}e^{\lambda_{2}\left(t\right)}\\ -b_{4}e^{\lambda_{1}\left(t\right)}+b_{4}e^{\lambda_{2}\left(t\right)}\; & b_{5}e^{\lambda_{1}\left(t\right)}-b_{6}e^{\lambda_{2}\left(t\right)} \end{array}\right]$$

and

$$\begin{array}{c} b_{1}=\frac{\left(K_{2}+\lambda_{1}\right)}{\left(\lambda_{1}-\lambda_{2}\right)},\;b_{2}=\frac{\left(K_{2}+\lambda_{2}\right)}{\left(\lambda_{1}-\lambda_{2}\right)},\;b_{3}=\frac{1}{\left(\lambda_{1}-\lambda_{2}\right)},\\ b_{4}=\frac{K_{1}}{\left(\lambda_{1}-\lambda_{2}\right)},\;b_{5}=\frac{\lambda_{1}}{\left(\lambda_{1}-\lambda_{2}\right)},\;b_{6}=\frac{\lambda_{2}}{\left(\lambda_{1}-\lambda_{2}\right)} \end{array}$$

where the eigenvalue is calculated as

(71) $$\lambda_{1,2}=\frac{-K_{2}\pm \sqrt{K_{2}^{2}-4K_{1}}}{2}$$

Considering that condition **C2.** in Proposition 3 emphasizes that the gain parameters of the proposed control law $u_{1}$ are taken to ensure that the eigenvalues of subsystem (70) lie in the open left-half plane. Therefore, both $\xi_{1,1}$ and $\xi_{1,2}$ states of WMR will never cross the zero. In addition, **C3.** restricts the initial condition of $\xi_{1,1}\left(0\right)\neq 0$ to avoid singularity during the initial transient in (68b). Hence the solution of Equation (68b) will remain bounded ∀t⩾0. □

Posture stabilization of a nonholonomic mobile robot (69a) and (69b) can be attained by employing the backstepping SMC $u_{1}$ from Equations (32) and (65) and $u_{2}$ from Equations (38) and (39) (by setting all reference signals to zero).

### 7.4. Output Feedback Control

The closed-loop system (60a)–(60c) is further designed under output feedback control using the notation described in Equations (47a) and (47b) and Equations (48a) and (48b). The proposed full-order high gain observer for differential drive WMR are

(72a)η^˙=ξ^1,2ξ^2,1+Lη(ζ−η^)(72b)ξ^˙1,1=ξ^1,2+(h1,1/ϵ1)(y1−ξ^1,1)(72c)ξ^˙1,2=α¯1(η^,ξ^)+Γ¯01(η^,ξ^)+β¯01(η^,ξ^)τ+(h1,2/ϵ12)(y1−ξ^1,1)(72d)ξ^˙2,1=ξ^2,2+(h2,1/ϵ2)(y2−ξ^2,1)(72e)ξ^˙2,2=α¯2(η^,ξ^)+Γ¯02(η^,ξ^)+β¯02(η^,ξ^)τ+(h2,2/ϵ22)(y2−ξ^2,1)

## 8. Simulation Results

This section presents numerical simulations of robust trajectory tracking and posture stabilization of differential drive WMR using state feedback and output feedback backstepping sliding mode control (BSMC) under bounded parameter uncertainties and external disturbance. The nominal values of the physical parameters of the mobile robot are as follows: r=0.05 m, m=4 kg, 2L=0.27 m, d=0.05 m, and I=2.5 kg.m^2^. During the simulation, parameter uncertainty on all physical parameters are assumed up to 20% of their nominal values, and an external disturbance of $\tau_{d}={\left[5\sin\left(3t\right),\;5\sin\left(3t\right)\right]}^{T}$ is applied. The control parameters for trajectory tracking are chosen as follows: $k_{1}=3.5$, $k_{3}=4$, $k_{4}=20$, $a_{1}=a_{2}=0.2$, $\gamma_{1}=10$, $\gamma_{2}=100$, $\mu_{1}=\mu_{2}=0.1$, $h_{1,1}=2$, $h_{1,2}=3$, $h_{2,1}=6$, $h_{2,2}=11$, $L_{\eta }=5$, $\epsilon_{1}=\epsilon_{2}=0.01$, $x_{c}\left(0\right)=0.3$, $y_{c}\left(0\right)=-0.7$, $\theta \left(0\right)=45^{\circ }$, ${\hat{x}}_{c}\left(0\right)={\hat{y}}_{c}\left(0\right)=0$, $\hat{\theta }\left(0\right)=57^{\circ }$, and ${\dot{\theta }}_{r}$ = 5 (rad/s).

The control parameters *a* and γ in backstepping SMC $u_{1}$ (32) and $u_{2}$ (38) can be calculated using the inequalities defined by Equations (23a) and (23b). By choosing the small value of μ in backstepping SMC, we can recover the performance of discontinuous SMC. However, if μ is excessively small, it may lead to chattering. Hence, a value of μ = 0.1 is selected to mitigate the occurrence of chattering effects. The controller gain parameters $k_{1},k_{3}$, and $k_{4}$ are chosen to ensure asymptotic stabilization of WMR towards the origin. The observer gain parameters $h_{i,1}$ and $h_{i,2}$ are selected such that the polynomial $s^{2}+h_{i,1}s+h_{i,2}$ is Hurwitz. Furthermore, it is desirable to have a small value of $\epsilon_{i}$ in the high gain observer, to ensure that estimation errors quickly converge to zero.

**Figure 2:**
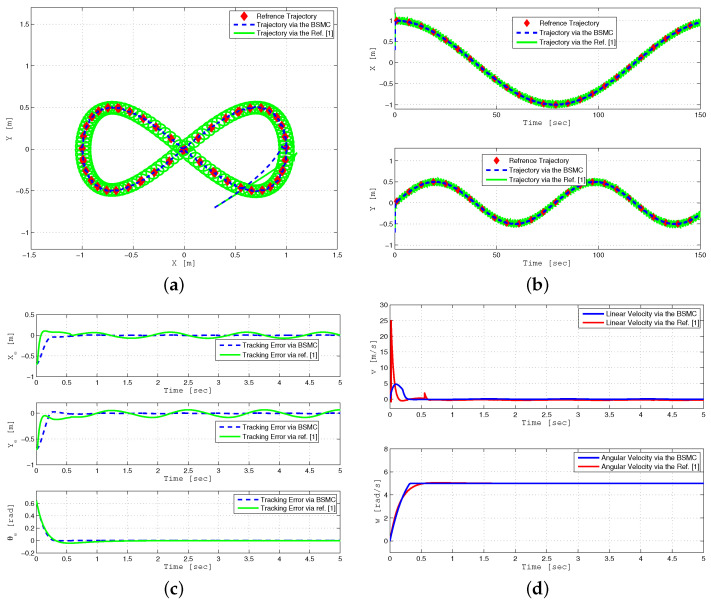
Lemniscate curve Trajectory tracking performance of WMR for BSMC (dashed line) and backstepping controller in reference [1] (solid line) under parameter uncertainties and external disturbance (**a**–**d**).

The first simulation displays the robust trajectory tracking of a WMR using the state feedback control, as illustrated in Figure 2. The performance of the proposed backstepping SMC is compared with the approach presented in [1] under bounded parameter uncertainties and external disturbances. In this simulation, a lemniscate curve reference trajectory is selected as follows:

$$x_{r}\left(t\right)=sin\left(0.04t\right),\;\;y_{r}\left(t\right)=sin\left(0.08t\right)$$

In particular, Figure 2a,b display the trajectory tracking of a mobile robot to a lemniscate curve reference trajectory. The convergence of tracking errors ($x_{e},y_{e},\theta_{e}$) to zero is presented in Figure 2c. It can be observed that the proposed robust backstepping sliding mode control minimizes tracking errors compared to previous backstepping control [1], which cannot efficiently deal with these uncertainties.

**Figure 3:**
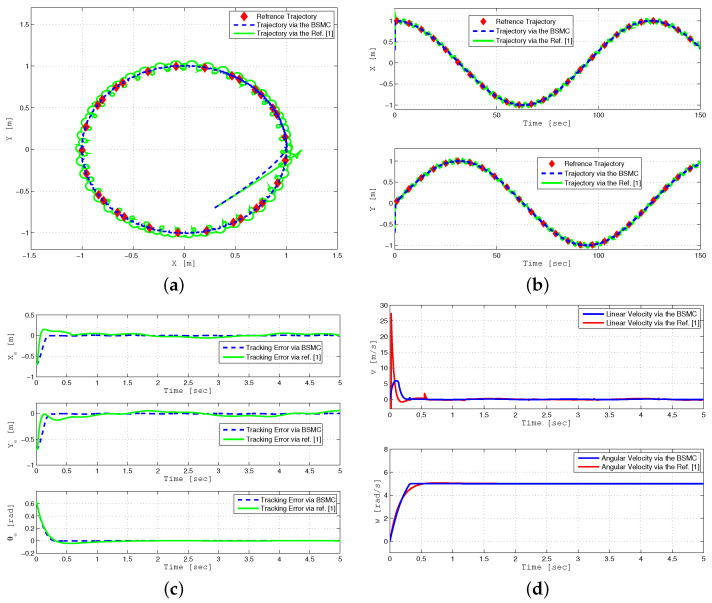
Circular trajectory tracking performance of WMR for BSMC (dashed line) and backstepping controller in reference [1] (solid line) under parameter uncertainties and external disturbance (**a**–**d**).

Figure 3a–d display the performance of the tracking controller in the presence of white noise. The presented results demonstrate that the suggested backstepping SMC control accurately tracked the reference trajectory in the x−y plane compared to the previous control approach described in [1]. Furthermore, Figure 3c shows that the tracking errors ($x_{e}$, $y_{e}$, and $\theta_{e}$) asymptotically converge to zero even in the presence of white noise using the proposed controller.

**Figure 4:**
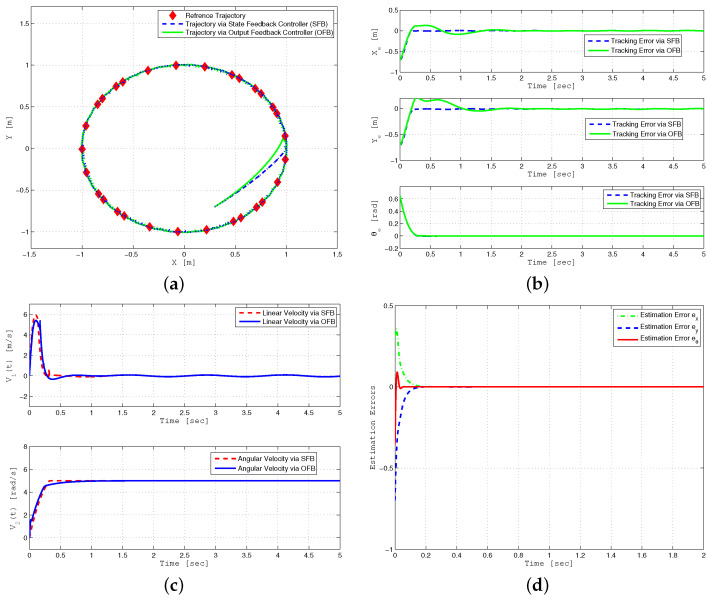
Trajectory tracking performance analysis of WMR using SFB and OFB controllers under parameter uncertainties and external disturbance (**a**–**d**).

The second simulation (Figure 4) compares the performance of trajectory tracking under state feedback (SFB) and output feedback (OFB) controllers. For simulation purposes, a circular trajectory is defined as:

$$x_{r}\left(t\right)=cos\left(0.05t\right),\;\;y_{r}\left(t\right)=sin\left(0.05t\right)$$

**Figure 5:**
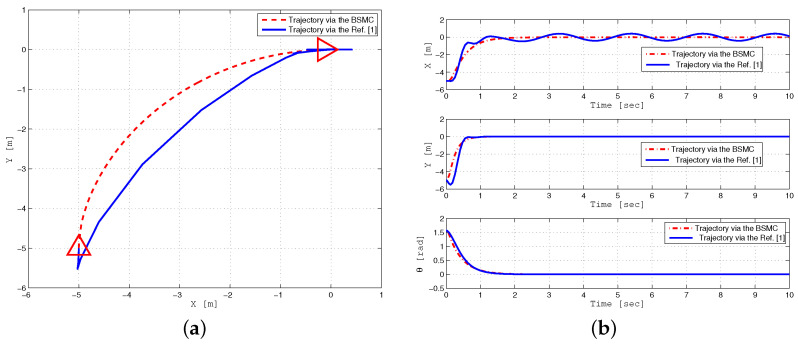
Posture stabilization performance of WMR for BSMC (dashed line) and backstepping controller in reference [1] (solid line) under parameter uncertainties and external disturbance (**a**,**b**).

**Figure 6:**
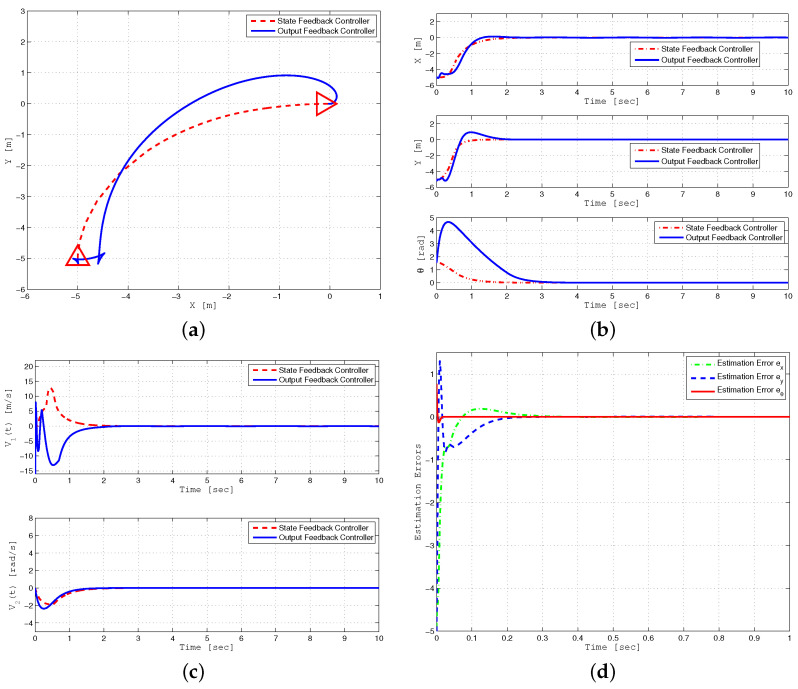
Posture stabilization performance analysis of WMR using SFB and OFB controllers under parameter uncertainties and external disturbance (**a**–**d**).

Figure 4a shows that trajectory tracking under output feedback with estimated states approaches the response obtained under the state feedback control, despite parameter uncertainties and time-varying external disturbance. Asymptotic convergence of the tracking error, i.e., $x_{e}=x-x_{r}$ to zero is presented for both scenarios in Figure 4b. The performance of linear and angular velocities using both control techniques is depicted in Figure 4c. The estimation errors $e_{x}=x-\hat{x}$, $e_{y}=y-\hat{y}$, and $e_{\theta }=\theta -\hat{\theta }$, under the output feedback design are displayed in Figure 4d, showing that the estimation errors converge to zero quickly. Furthermore, Table 2, compares the performance of the proposed controller in comparison with the previous controller presented in [25]. Simulation results illustrated in Figure 4 are compared with simulation results (Figures 2–7) presented in [25].

The control parameters selected for robust posture stabilization are as follows: $k_{1}=2.5$, $k_{3}=8$, $k_{4}=7$, $a_{1}=a_{2}=0.7$, $\gamma_{1}=12$, $\gamma_{2}=150$, $\mu_{1}=\mu_{2}=0.1$, $h_{1,1}=6$, $h_{1,2}=13$, $h_{2,1}=2$, $h_{2,2}=6$, $L_{\eta }=12$, $\epsilon_{1}=\epsilon_{2}=0.01$, $x_{c}\left(0\right)=-5$, $y_{c}\left(0\right)=-5$, $\theta \left(0\right)=90^{\circ }$, ${\hat{x}}_{c}\left(0\right)={\hat{y}}_{c}\left(0\right)=0$, and $\hat{\theta }\left(0\right)=45^{\circ }$. Figure 5 displays the robust posture stabilization of a mobile robot using state feedback control. The performance of posture stabilization using the proposed backstepping sliding mode control (BSMC) is compared with the backstepping controller [1] under bounded parameter uncertainties and external disturbance. As demonstrated in these graphs, the proposed control law successfully achieves posture stabilization compared to the previous approach [1].

Finally, Figure 6a displays the posture stabilization performance analysis under output feedback and state feedback control schemes. Figure 6b,c show that the output feedback controller based on HGO can recover the state feedback performance by converging the estimated state to the true state rapidly even in the presence of parameter uncertainties and time-varying external disturbance. Figure 6d displays the estimation errors of posture variables, i.e., $e_{x}=x-\hat{x}$, as well as the linear and angular velocities under output feedback control. It illustrates that estimation errors quickly converge to zero due to a small value to ϵ=0.01 in the high gain observer.

## 9. Conclusions

This paper focuses on the design of a robust output feedback control that is simultaneously valid for both trajectory tracking and stabilization for a class of MIMO underactuated nonholonomic systems. The control design is based on kinematic and dynamic models in a globally defined normal form. We also consider that the nonholonomic system is under the influence of bounded model uncertainties and disturbances and in the absence of velocity measurements. The control and stabilization of these systems are widely regarded as the most difficult benchmark problems because of various inherent difficulties. These include: (i) it is not exactly feedback linearizable (input-state linearizable) because of nonholonomic constraints, (ii) obtaining a cascade normal form structure in the strict feedback form is not always possible, (iii) internal dynamics of nontriangular normal form are non-affine in control, i.e., highly nonlinear, and (iv) zero dynamics of the system are not minimum phase. The significant contribution of this research is the formulation of a generalized normal form using an input–output feedback linearization and change in coordinates approach that provides ease in designing (1) unified backstepping sliding mode control solutions for both stabilization and trajectory tracking subject to external disturbances and parameter uncertainties and (2) full-order HGO for the estimation of the derivative of output functions and internal dynamics. Moreover, a full-order high-gain observer and the backstepping sliding mode control are integrated to synthesize a robust output feedback controller. The proposed backstepping SMC reduces or eliminates the chattering phenomenon by dividing the control law into continuous and switching components, i.e., the high-slope saturation function. A differential drive type (2,0) WMR is used as an example to show the performance of the proposed controller. The simulation results illustrate that the suggested control law is robust against the bounded uncertainties, and the output feedback controller with estimated states can retrieve state feedback control performance (trajectory tracking and posture stabilization) even in the presence of such uncertainties. Therefore, both asymptotic trajectory tracking and posture stabilization are achieved in semi-global regions, with nonzero initial condition of the heading angle compared with nonzero initial condition of desired velocities in previous research.

## 10. Future Work

One promising area of potential future research in the field of nonholonomic systems is the design of robust control methodologies specifically tailored for systems affected by unmatched uncertainties. Another possible direction for future work is the study of formation/consensus control of wheeled mobile robots, as cooperative control of these vehicles and its real-world implementation continue to be active areas of investigation.

## Figures and Tables

**Table 1:** Table of nomenclature.

Symbol	Meaning
*q*	generalized coordinates
J(p)	kinematic constraints matrix
ϑ(t)	velocity vector
τ	input torque vector
ρ	relative degree
η	internal dynamics
ξ	external dynamics
*s*	sliding surface
a,μ	positive constants
$u_{1eq}$	equivalent control input
*k*	controller gain
$h_{1,1},h_{1,2}$	observer gains
sat(x)	saturation function
*K*	class K functions
g(0)	substituting x=0 in input transformation matrix g(x)

**Table 2:** Output feedback trajectory tracking performance analysis.

Parameter	Proposed Controller	Previous Method [25]
Tracking position errors (m)	≈0.001	≈0.021
Tracking errors convergence time (s)	≈ 2	≈5
Estimation error $e_{v_{1}}=v_{1}-{\hat{v}}_{1}$	zero	not equal to zero
Estimation error $e_{v_{2}}=v_{2}-{\hat{v}}_{2}$	zero	not equal to zero

## Data Availability

Data are contained within the article.

## Abbreviations

The following abbreviations are used in this manuscript:

SMC	Sliding mode control
HGO	High gain observer
WMRs	Wheeled mobile robots
MIMO	Multi-inputs multi-outputs
BSMC	Backstepping sliding mode control
SFB	State feedback
OFB	Output feedback
